# Supplementary material for: Neck musculoskeletal model generation through anthropometric scaling
Source: PLoS One. 2020 Jan 28;15(1):e0219954. doi: 10.1371/journal.pone.0219954 (PMC6986765; doi:10.1371/journal.pone.0219954)
Supplement: Attachment — Submitted filename: Response To Reviewers.pdf [file pone.0219954.s002.pdf]

Responses to reviewers' comments

Dear Dr. Bernadette Murphy,

Thank you for considering our manuscript for publication in PLOS ONE.

We want to thank both reviewers for their comments and suggestion, which are very helpful for improving and strengthening our paper. Below we addressed each of their comments with "Response: …" in red text. In the submitted revision, corresponding changes in the text are marked with red color as well.

Sincerely,

Xianlian Zhou, PhD

Reviewer #1: Comments:

===================

This is a very well written paper that provides a rather elegant and useful solution to generate subject-specific models of the neck. The use of a bilevel optimisation is a great idea. Also the discussion and description of the improvements with respect to previous methods is very clear and valid. However there are several points which appear less clear within the paper:

The authors would like to thank the reviewer for their positive feedback.

Line 67 - Please provide little more info on the ANSUR II 3D database, and briefly explain why this approach can make the difference in addressing the previously highlighted challenges.

Detail has been added on the ANSUR II database, for example, in the section on "50th percentile anthropometric models" (lines 110-126). In addition, we added a line to highlight the strength of the approach used – "This allows the generation of personalized neck musculoskeletal models with realistic strength and anthropometry that are required to address research questions that consider the effect of body size and gender on predicting *in vivo* neck loadings." (Lines 64-67)

Line 82. Please provide more info on AMG software and how it can be used. More info is provided later on (Line 124), but an initial clarification would be helpful.

The following line has been added – "The AMG software is in-house developed software (by CFD Research) that generates anthropometric models using Principal Component Analysis (PCA)." (Lines 81-83) And additional background on the method used were added in the section of "50th percentile anthropometric models" (Lines 110-126).

Line 123. Please clarify what ‘synthesised’ mode the authors are referring to (I guess the ANSUR II model within the AMD).

It has been clarified that this refers to the models that are synthesized using the AMG software. (Line 139)

Line 129. Please clarify what ‘manually’ means? Was the optimal fir chosen visually?

Yes, this optimal fit was chosen visually. Extra detail has been added on the process of manual scaling. – “The manual scaling was done by scaling separate body segments to obtain a visually close-fitting match.” (lines 147-148)

Fig 3. Please clarify the colour coding and its relation with density (why different tones of blue and red?)

The different tones in blue and red just identify the different body parts, there is no specific color coding. This has been clarified in the figure legend.

Line 218. Please clarify the terminology used within the whole paper in relation to the different models used. Is the original neck model, the Vasavada’s (+ hyoid) model?

Correct, the original neck model is Vasavada’s (+hyoid model). This is described already in detail in paragraph 1. Scaling of original neck model to 50th percentile male (and female) anthropometry. We therefore reference this paragraph.

Line 238. Please clarify what the authors mean with a ‘inner static muscle optimisation’ as it can be mixed up with a classic static optimisation procedure (which would need to solve for the muscle redundancy problem). Therefore the authors should clarify how the muscle activations were set to generate the target joint torques (e.g. max muscle activation). Otherwise the authors should clarify how the muscle redundancy problem was solved within the bilevel optimisation (e.g. minimising sum of muscle forces?). How did the optimiser ensured a physiological muscles activation?

Details have been added to explain the inner static optimization process. The muscle redundancy problem was solved using an objective function that minimizes a weighted sum of muscle effort and residual torques. (Lines 271-284)

Also, how did the authors ensured symmetry between the right and left side of the model? The

optimiser might find asymmetrical solutions. If this is dealt with as described in Lines 298-300, it should be better clarified beforehand.

For the optimization of the 50th percentile models, we ensured symmetry between left and right for all muscles. We added a line at the end of the muscle optimization section to explicitly state: "During the optimization, the left-right symmetry of all muscles was enforced through equality constraints of muscle parameters". (Lines 314-315)

However, during interactive scaling, we cannot guarantee symmetry because the skin mesh may not be perfect symmetric, which causes muscle paths to differ between left and right. Such asymmetric results were dealt with as is described in line 338-341.

The authors should move the description of the optimisation methods (Line 262-268) before the description of the objective function.

Thank you for this suggestion, we have moved this paragraph before the description of the objective function.

Line 250. Please clarify what the final objective function formula (J + Jp ?)

Correct, this is J+Jp, this has now been clarified. (Line 293)

Line 244-250. Unfortunately this explanation is not clear and I require the authors to provide the optimisation results, and a breakdown of the optimal neck muscles force across the different degrees of freedom.

As pointed out by the review, the inner static optimization was not explained clearly, which might have caused confusion here. Now as we added detailed explanation of the static optimization process, the meaning of unnecessary high strength and how it was detected and treated shall become clear. Since the inner static optimization is standard, we try to avoid adding excessive details to the paper. In addition, we changed "In this case" to "Therefore" to indicate the penalty term is always there during the optimization.

Line 271-274. The authors make a good point of using Zheng's model as the previous model was based on cadaveric data. However here it becomes clear that the muscles volume distribution is still based on the Vasavada' model. Is there any limitation in relation to this approach?

The volumes of the groups are based on Zheng's model, this however does not have a distribution of the individual muscle subvolumes within these groups. These individual

subvolume proportions are based on the original Vasavada model. This is now emphasized in the text. (Lines 311-315)

Lines 309-327. One of the main limitations of Vasavada's model is related to the neck muscles paths, as they are not anatomically realistic, especially considering the superficial muscles (traps). The authors are proposing a scaling factors mainly based on the geometrical position of different nodes, which can be rather different from the anatomical equivalent. Although I understand and appreciate the rigorous attempt to solve this problem, the error in the muscle paths and nodes position might be greater than a rough 3D scaling. Previous use of wrapping surfaces was attempted, and it might help to improve such scaling. Please comment on this.

As we pointed out in the discussion, muscles can be modeled as running closer to the body using wrapping objects or via points as have been done by Suderman et al. (Lines 470-472). However, these wrapped muscle models can be very sensitive to wrapping object or via point kinematics and inter-individual differences in muscle paths and joint kinematics. Using wrapping objects increases the complexity of the model and often makes scaling much harder. Scaling of these wrapping objects together with muscle nodes can often result in erroneous muscle paths and moment arms, which defeats the purpose of using the wrapping objects in the first place.

Line 360-362. This should be included in the methods.

This was put in the results section because we discovered the need to add these additional rectus capitis muscles only after analyzing the initial results. If we put these lines into the method section, we feel it lacks context.

Reviewer #2: Comments:
===================

In review of "Neck Musculoskeletal Model Generation through Anthropometric Scaling" a paper seeking to accurately estimate neck loads with a scaling parameter. The topic is definitely worthy of study and the ability to scale biomechanical models to the individual is also a valuable contribution to the literature.

The authors would like to thank the reviewer for their positive feedback.

Intro: There is no need to start this paper with a description of neck pain. Readers ought to be aware of the applications of biomechanically model. I would rather like to see an introduction into the main issues this paper and work will address perhaps in the broader context of the literature. (ie how does this paper build on neck models and why is that important - focus on why what you have done is important and don't distract from this with a discussion on neck pain that is really part of the work which will follow the development of the model)
Finally, the intro stops abruptly - summarize the introduction rather then leaving the reader hanging on your last thoughts.

Thanks for the suggestion. We have simplified the first paragraph to de-emphasize neck pain or injury and introduce the need for estimating *in vivo* neck loading. We have also added an extra line to the introduction to make the end less abrupt and explain the goal of this study:

"This allows the generation of personalized neck musculoskeletal models with realistic strength and anthropometry that are required to address research questions that consider the effect of body size and gender on predicting *in vivo* neck loadings." (Lines 64-67)

Methods:
More details about the AGM software would be helpful it seems this study heavily relies on this work which means is also is subject to the assumptions made in this work - what are those assumptions?

Thanks for the suggestion. Detail on the ANSUR II database, principal component analysis (PCA), and AMG software has been added, for example, in the section on "50th percentile anthropometric models" (Lines 110-126), to help readers understand the underlying approach and assumptions. Additional details and discussion can be found in the referred article (Zhou, et al. IJDH, 2016).

Is the female scaled to the 50th percentile and then those values extracted from the male data to match the size of the 50th percentile female? It is unclear when you use "male (female)" what exactly you are doing and mean by this. (Okay this comes at the end of the first section of the methods but is reference before it is fully described. Consider re-organizing). Also please explain what does gender specific differences mean?

We thank the reviewer for pointing this out and have re-organized the text to improve clarity. As explained in the last paragraph of the section on 50th percentile anthropometric models, the female musculoskeletal model was generated by manually scaling the original (male) model while incorporating gender specific differences. An extra line has also been added to clarify what we mean with gender specific differences – ", such as differences in mass and inertia distributions, as well as muscle strength with regards to anthropometry". (Lines 185-186)

Prove to the reader the optimization techniques are not simply making the math work and the model remains physiologically meaningful.

The reviewer makes a good point here. The optimization method is a bi-level optimization process that utilizes an inner static optimization to solve muscle forces. In the section "Optimization of maximum isometric muscle force", we now added detailed explanation of the static optimization process, the meaning of unnecessary high strength, and why it is important to detect and treat it to make the model physiologically reasonable. In addition, the bounding constraints on muscle properties (such as the maximum isometric force) in the optimization (as in how much the muscle properties are allowed to vary) were set such that these properties remain physiologically relevant. (Lines 271-284)

Results:
"2. Neck Strength optimized models
he original male model was too weak in flexion, lateral bending, and axial rotation
and too strong in extension. " provide the reader with some magnitude to quantify this amount!

This is shown in the first two lines of table 1:

models.

	Extension (N)	Flexion (N)	Lateral bending (N)	Axial rotation (Nm)
**Original model**	255	66	109	7.4
**Male target literature**	254	122	173	11.2
[illegible]	[illegible]	[illegible]	[illegible]	[illegible]

The table shows flexion strength was 66 N in the model, compared to the average of 122 N reported in literature. Lateral bending was 109 N in the original model, compared to the average of 173 N reported in literature, and axial rotation strength was 7.4 Nm in the original model, compared to the average of 11.2 Nm reported in literature.

We have added a reference to table 1 after this sentence for clarity. And for clarity we have added the change in percentage difference from the target data from literature. (Lines 396-398)

"After optimization, the strength of the 50th percentile male was improved significantly for most directions. " quantify this amount for the reader.

See above about quantifying the amount. Per reviewer's suggestion, we have removed the word "significant".

Based on Table 4 - what model do you trust more and why? The values for some muscles (esp. flexion muscles) are very very different. stern_mast 86.1 N to 221.58 N. That seems like a major difference in prediction.

Thanks for pointing this out. Even though the muscle volumes of the original models are cadaver based, the strength of these models was just slightly more than half of what was reported in literature (see table 1). As a consequence of trying to generate the required flexion strength, the max forces of most flexor muscles were increased substantially, including the stern_mast. Since there is a large variance in reported muscle specific tension that determine the maximum muscle force along with PCSA, our muscle prediction is within specified force range that deemed physiologically reasonable. For applications in which the overall neck strength is important, we believe our new model will provide better prediction.

Also Rec Cap is a very small muscle and has a predicted force of 92.6 N compare this to Stern Mast originally predicted at 86.1 N.

The reviewer makes a good point that the strength of Rec Cap is high in the optimized model compared to other muscles. This was however required to obtain a realistic overall neck strength since our optimization considers all cervical joints from top to bottom. The Rec Cap muscle is particularly important in ensuring the strength on the top cervical joint. Since the Stern Mast in the optimized model is increased to 221.58, the Rec Cap strength in this model is still below 50%.

Was the model tested only in a neutral posture?

Yes, it was. There is insufficient data available on neck strength in different postures.

Discussion: If muscles had via points and smaller moment arms - how would this effect your predicitions - would this make muscle flexion force even higher for example?

It is possible some flexor muscles could have even higher force if some muscles are changed to have smaller moment arm. However, this is uncertain since the optimization considered all cervical joints and muscle coordination in resisting the external forces. Further, Suderman's work as well as Vasavada's earlier study (Vasavada et al., J Biomech, 2008, Defining and evaluating MRI-derived wrapping surfaces for spinal muscles), showed that the SCM is relatively straight and not as affected by wrapping as the extensors are.
We considered using neck models with muscles running closer to the body using wrapping objects or via points as have been done by Suderman et al. However, these wrapped muscle models can be very sensitive to wrapping object or via point kinematics and inter-individual differences in muscle paths and joint kinematics. It increases the complexity of the model and often makes scaling much harder.

Line 476 : In (8), it was – rephrase

We changed this to: "Zheng et al. (8) showed that …" (Lines 516-517)

Overall:
Refrain from using "significantly" if you are not citing a specific statistical difference. ie females have significantly smaller necks.

We have removed the word "significantly" where used inappropriately. We thank the reviewer for pointing this out.

Finally - limited information of the original method of force estimation is provided. More details on the methods of the original force estimation (prior to optimization) would be helpful - was this done?

Thanks for pointing this out. We added text to explain the method to determine the strength of the original model in the section “Optimization of maximum isometric muscle force”:

“The overall neck strength was computed by simulating the experimental conditions with increasing forces or (axial rotation) moment applied to the model until it can no long maintain the static neutral posture. For example, to determine the flexion strength, we applied an increasing force on the forehead and conducted static optimization to determine if the muscles can coordinate to generate the required joint torques.” (Lines 240-245)
